# Rural–urban disparity in community-based health insurance enrollment in Ethiopia: a multivariate decomposition analysis using Ethiopian Mini Demographic Health Survey 2019

**DOI:** 10.3389/fpubh.2024.1361793

**Published:** 2024-07-29

**Authors:** Yawkal Tsega, Hiwot Tadesse Alemu, Demiss Mulatu Geberu, Asebe Hagos, Melak Jejaw, Kaleab Mesfin Abera, Misganaw Guadie Tiruneh, Kaleb Assegid Demissie, Lakew Asmare, Abel Endawkie, Wubshet Debebe Negash, Amare Mesfin Workie, Lamrot Yohannes, Mihret Getnet, Nigusu Worku, Adina Yeshambel Belay

**Affiliations:** ^1^Department of Health Systems and Management, School of Public Health, College of Medicine and Health Sciences, Wollo University, Dessie, Ethiopia; ^2^Department of Health Systems and Policy, Institute of Public Health, College of Medicine and Health Sciences, University of Gondar, Gondar, Ethiopia; ^3^Department of Epidemiology and Biostatistics, School of Public Health, College of Medicine and Health Sciences, Wollo University, Dessie, Ethiopia; ^4^Department of Nutrition, Institute of Public Health, College of Medicine and Health Science, University of Gondar, Gondar, Ethiopia; ^5^Department of Environmental and Occupational Health and Safety, Institute of Public Health, College of Medicine and Health Science, University of Gondar, Gondar, Ethiopia; ^6^Department of Epidemiology and Biostatistics, Institute of Public Health, College of Medicine and Health Science, University of Gondar, Gondar, Ethiopia; ^7^Department of Human Physiology, School of Medicine, College of Medicine and Health Science, University of Gondar, Gondar, Ethiopia

**Keywords:** rural–urban, disparity, community-based health insurance, multivariate decomposition analysis, EMDHS 2019

## Abstract

**Background:**

In sub-Saharan Africa, achieving universal health coverage (UHC) and protecting populations from health-related financial hardship remain challenging goals. Subsequently, community-based health insurance (CBHI) has gained interest in low and middle-income countries, such as Ethiopia. However, the rural–urban disparity in CBHI enrollment has not been properly investigated using multivariate decomposition analysis. Therefore, this study aimed to assess the rural–urban disparity of CBHI enrollment in Ethiopia using the Ethiopian Mini Demographic Health Survey 2019 (EMDHS 2019).

**Methods:**

This study used the latest EMDHS 2019 dataset. STATA version 17.0 software was used for analyses. The chi-square test was used to assess the association between CBHI enrollment and the explanatory variables. The rural–urban disparity of CBHI enrollment was assessed using the logit-based multivariate decomposition analysis. A *p*-value of <0.05 with a 95% confidence interval was used to determine the statistical significance.

**Results:**

The study found that there was a significant disparity in CBHI enrollment between urban and rural households (*p* < 0.001). Approximately 36.98% of CBHI enrollment disparities were attributed to the compositional (endowment) differences of household characteristics between urban and rural households, and 63.02% of the disparities were due to the effect of these characteristics (coefficients). The study identified that the age and education of the household head, family size, number of under-five children, administrative regions, and wealth status were significant contributing factors for the disparities due to compositional differences between urban and rural households. The region was the significant factor that contributed to the rural–urban disparity of CBHI enrollment due to the effect of household characteristics.

**Conclusion:**

There were significant urban–rural disparities in CBHI enrollment in Ethiopia. Factors such as age and education of the household head, family size, number of under-five children, region of the household, and wealth status of the household contributed to the disparities attributed to the endowment, and region of the household was the contributing factor for the disparities due to the effect of household characteristics. Therefore, the concerned body should design strategies to enhance equitable CBHI enrollment in urban and rural households.

## Background

Ensuring everyone has access to essential health services is a key commitment of the 2030 Agenda for Sustainable Development, which affirms health as a basic human right. The most effective way to achieve the motto of “leave no one behind” is by providing universal health coverage (UHC). It is aimed to ensure that people can access high-quality healthcare without facing financial risks due to healthcare expenditures (1–4).

Although the UHC movement has been gaining support worldwide (1), in 2021, nearly half of the global population—4.5 billion people—did not have access to essential healthcare services. In 2019, nearly 2 billion people experienced financial hardship because of their healthcare expenditures, with 344 million of them being extremely poor (1). Achieving UHC for most countries in sub-Saharan Africa has been a challenging agenda so far. This has led to more interest in community-based health insurance (CBHI), a health insurance scheme that has been established for workers of non-formal sectors in many countries such as Ethiopia (2, 3, 5–7).

Ethiopia is among the lowest spenders on health systems in Africa. In 2016, only 1.4% of its gross domestic product (GDP) was allocated to health (8). The country also fell short of the 15% target set by the Abuja Declaration, allocating only 11.1% of the total government budget to health in 2014 (9–11). The latest National Health Account report revealed that Ethiopia spends only 4.7% of its GDP on health, which is lower than the target recommended by the World Health Organization. Additionally, people had to pay 30% of the total healthcare costs directly from their own pockets, which is the highest rate in the world and in Africa (12). Moreover, accessing essential healthcare remains a significant challenge for achieving UHC in Ethiopia, especially for underprivileged people living in rural areas (2, 3, 6, 7, 13, 14).

CBHI is a type of volunteer health insurance that aims to provide financial protection and access to quality healthcare for the poor, vulnerable populations, and informal sector workers. Ethiopia launched CBHI in 2011 with a vision of reaching 80% of districts and 80% of its population by 2020 (14–20). However, despite the government’s efforts to expand CBHI to all districts and studies conducted on different aspects of CBHI including spatial analysis (7), to the best of authors knowledge, evidence is lacking on the assessment of rural–urban CBHI enrollment disparity and the factors that contributed to the disparities using the logit-based multivariate decomposition analysis (14, 18–20). Therefore, this study aimed to assess the disparities in CBHI enrollment between rural and urban households and identify the factors contributing to these disparities due to endowments (E) and coefficients (C) by conducting a multivariate decomposition analysis of the EMDHS 2019 data. This study used a multivariate decomposition analysis model because it provides distinct advantages over spatial analysis, particularly in its ability to identify and quantify the contribution of individual variables on the outcome variable disparity between groups and urban and rural dimensions in this study. While the spatial analysis is adept at identifying patterns and clusters within geographical data, the multivariate decomposition analysis provides a deeper understanding of the underlying factors determining these patterns. This method allows for a more nuanced analysis of trends and can inform targeted interventions by pinpointing the exact factors that contribute to changes in outcomes over time or between two distinct groups, making it a powerful tool for policy-making and strategic planning in various fields of research (21, 22). Therefore, this study aimed to answer the following two main research questions;

1. Was there an urban–rural disparity in the CBHI enrollment rate in Ethiopia in 2019?
2. If a disparity existed, what factors were contributing to the disparity?

## Methods

### Data sources, study setting, and populations

Ethiopia is a landlocked country in the Horn of Africa (3°–14° N and 330–48° E), with nine regional states (Tigray, Afar, Amhara, Oromia, Somalia, Benishangul-Gumuz, Southern Nations, Nationalities, and Peoples’ Region (SNNPR), Gambella, and Harari) and two city administrations (Addis Ababa and Dire Dawa city administrations) in 2019 (23). The source of data for this study was the recent EMDHS 2019 dataset. The survey provides nationally representative data on a wide range of demographic and health indicators (24, 25). The survey used a two-stage cluster sampling technique stratified in nature, wherein enumeration areas (EAs) were the primary units and households were the secondary units (26). The dataset has been made freely available on the Internet for academics and researchers. The source population for this study was all households in Ethiopia. We excluded households who had missing information for CBHI enrollment and/or any of the explanatory variables of interest. After all, the final sample size comprised 8,663 households, of which 2,645 households were from urban areas and 6,018 households were from rural areas in this study.

### Study variables and measurements

#### Outcome variable

In this study, the outcome variable was CBHI enrollment. It was categorized as either “Yes” (labeled as 1) if enrolled for CBHI or “No” (labeled as 0) if the household did not enroll for CBHI.

#### Stratifying variable

Place of residency was the key independent variable used to stratify households by their CBHI enrollment status and was categorized as urban (labeled as “0”) or rural (coded as “1”).

#### Explanatory variables

Explanatory variables, known to be associated with CBHI enrollment from previous literature studies, were extracted from EMDHS 2019. These variables are the educational level of the household head (ordinal categorical variables with categories such as “no education”, “primary education”, “secondary education”, and “higher education”), wealth index (ordinal categorical variables with “poor,” “middle,” and “rich” category), age of household head, sex of household head, family size, and region of the household. The explanatory variables were categorized after consulting related previous literature (3, 4, 7, 15). All explanatory variables with their respective categories are presented in Table 1.

**Table 1:** List of extracted explanatory variables for urban–rural disparity in CBHI enrollment with their respective categories from the EMDHS 2019.

S/No	Variables	Category
1.	Region	Agricultural, pastoralist, city administrations
2.	Sex of household head	Male, female
3.	Age of household head	≤24, 25–64, ≥65
4.	Education level of household head	No education, primary, secondary, and higher
5.	Family size	≤4, 5–6, ≥7
6.	Number of under 5 children	No child, 1–2, ≥3
7.	Wealth index	Poor, middle, rich

### Statistical analysis

#### Descriptive statistics and chi-square test

STATA version 17.0 statistical software was used for data analysis. The STATA survey setting (svyset) command was used to control the complex clustering effect. The sociodemographic characteristics of the household heads and other variables were analyzed using descriptive statistics and were summarized as weighted frequencies and percentages. Pearson’s chi-square test was used to assess the association between CBHI enrollment and the explanatory variables. The explanatory variables having significant association (*p*-value <0.05) with CBHI enrollment during the chi-square test were included in a multivariate decomposition analysis.

#### Multivariate decomposition analysis

To estimate the observed disparities of the CBHI enrollment between rural and urban households, we used the logit-based multivariate decomposition analysis method (27). Multivariate decomposition is commonly used in social research to quantify the contributions to group differences in average predictions from multivariate models. It helps to divide the components of a group difference into those attributable to compositional differences (such as variations in characteristics or endowments) and those attributable to differences in the effects of characteristics (such as variations in returns, coefficients, or behavioral responses) (28).

Multivariate decomposition is useful to analyze disparities in health variables, such as race, sex, and other groupings. Although the method was originally designed for use in decomposing labor market outcomes between different groups, it has become useful in stratifying the outcome of health variables by various types of groups. This technique has also been applied in evaluating a given health variable across residencies, both urban and rural. The current study used the multivariate decomposition technique to decompose the difference in the CBHI enrollment for rural and urban areas of Ethiopia using the user-written mvdcmp STATA command (27, 29).

This method is appropriate because the difference in the observed CBHI enrollment rate between urban and rural households can be due to the difference in the distribution of the characteristics (compositional differences) and the effects of these characteristics (coefficient) or the interaction between the two components.

## Result

### Descriptive analysis and chi-square test

Table 2 shows the descriptive analysis of study participants and the association between explanatory variables with the outcome (i.e., CBHI enrollment) and equity-stratifying (i.e., place of residence) variables using the Pearson chi-square test. The overall weighted rate of CBHI enrollment in Ethiopia was 28.09% [95%CI, 26.68–29.58%] in 2019. Of the participants, approximately 68.04% were rural inhabitants residents without CBHI enrollment, and 80.60% were urban inhabitants without CBHI enrollment. Moreover, 77.93% of the households had male heads, with 47.38% having no education and 7% having higher education. Regarding the wealth status of the households, more than one-third (36.16%) and approximately half (44.50%) of the households were poor and rich, respectively (Table 2).

**Table 2:** Descriptive analysis of explanatory variables for CBHI enrollment disparity between urban and rural households using the EMDHS 2019.

Variables	Category	CBHI enrollment(*n* = 8,663)		Residence(*n* = 8,663)	
		Yes (*n*/%)	No (*n*/%)	Urban (*n*/%)	Rural (*n*/%)
Place of residence	Urban	517(19.40)	2,147(80.60)		
	Rural	1,917(31.96)	4,082(68.04)		
CBHI enrollment	Yes			517(19.40)	1,917(31.96)
	No			2,147(80.60)	4,082(68.04)
Sex of household head	Male	1,975(29.26)	4,776(70.74)	1,835(27.18)	4,916(72.82)
	Female	459(24.02)	1,453(75.98)	829(43.37)	1,083(56.63)
Household head education	No education	1,338(32.72)	2,752(67.28)	796(19.46)	3,294(80.54)
	Primary	834(27.16)	2,236(72.84)	968(31.52)	2,102(68.48)
	Secondary	178(20.37)	694(79.63)	510(58.52)	362(41.48)
	Higher	76(12.65)	525(87.35)	379(62.96)	223(37.04)
Age of household head	≤24	98(15.04)	553(84.96)	326(50.20)	324(49.80)
	25–64	1,917(28.36)	4,842(71.64)	2,044(30.24)	4,715(69.76)
	≥65	402(33.86)	784(66.14)	277(23.32)	909(76.68)
Family size	≤4	1,151(26.47)	3,196(73.53)	1,627(37.43)	2,720(62.57)
	5–6	780(33.05)	1,620(66.95)	645(26.64)	1,775(73.36)
	≥7	484(25.52)	1,412(74.48)	392(20.68)	1,504(79.32)
Number of under-five children	No child	1,239(28.24)	3,148(71.76)	1,567(35.71)	2,821(64.29)
	1–2 children	1,155(29.18)	2,802(70.82)	1,035(26.16)	2,921(73.84)
	≥3 children	40(12.69)	279(87.31)	62(19.44)	257(80.56)
Wealth status	Poor	786(25.10)	2,346(74.90)	258(8.23)	2,875(91.77)
	Middle	638(38.08)	1,037(61.92)	127(7.58)	1,548(92.42)
	Rich	1,010(26.20)	2,845(73.80)	2,279(59.12)	1,576(40.88)

Percentage was calculated from row total (percentage = cell value/row total) for each variable, CBHI enrollment and place of residence. The percentage and frequency were weighted using the weighting variable, which was taken from the EMDHS 2019 dataset.

**Figure 1:**
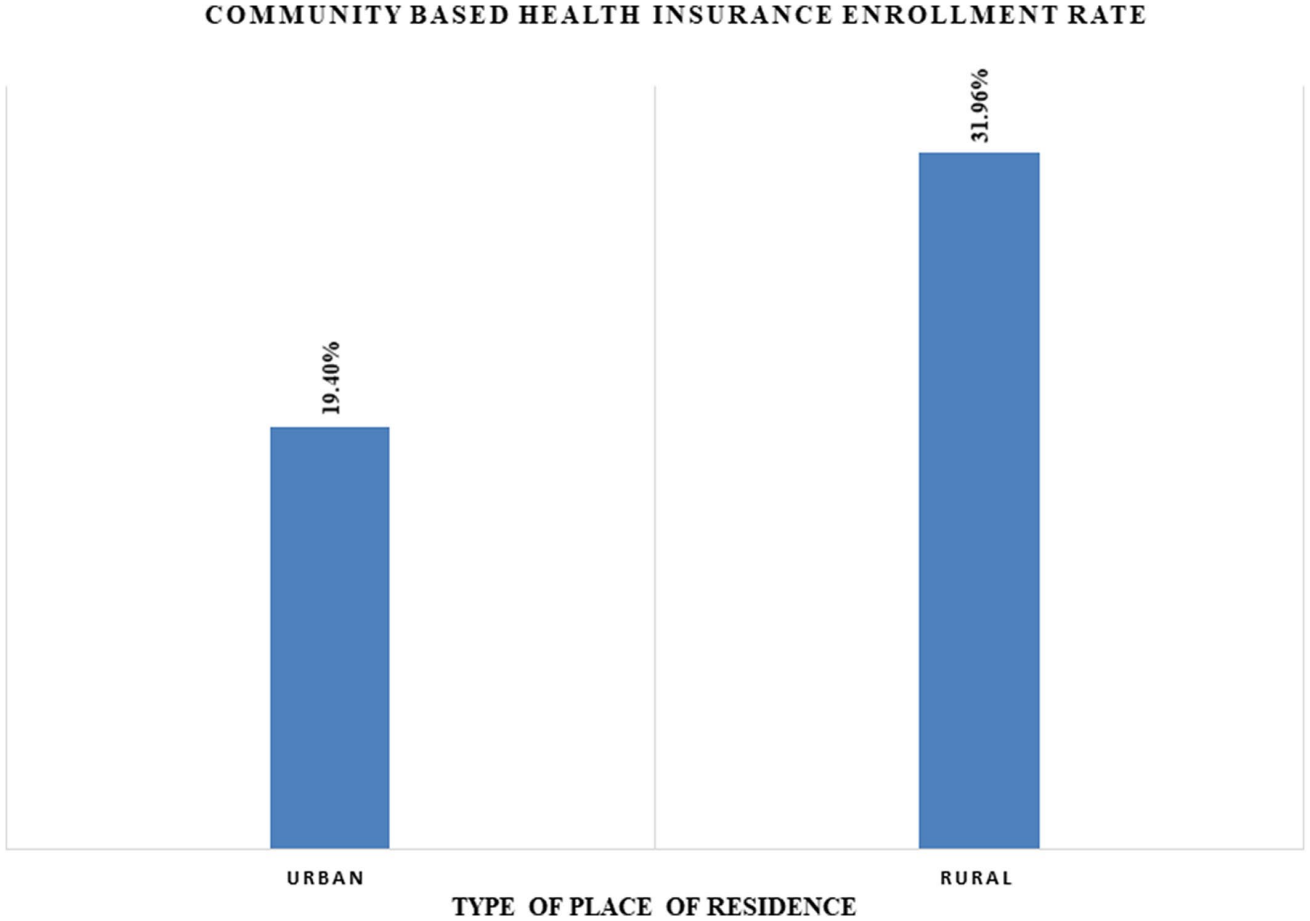
The CBHI enrollment rate across places of residence using the EMDHS 2019.

Figure 1 shows the CBHI enrollment rate across places of residence, such as urban and rural households. Nearly 32% of the households in the rural area were enrolled for CBHI, whereas 19.40% of the households in the urban area were enrolled for CBHI in 2019 in Ethiopia, indicating that rural households were more likely to be enrolled for CBHI in 2019.

### Multivariate decomposition analysis

Table 3 shows the overall decomposition analysis of the urban–rural CBHI enrollment rate disparity. This disparity was classified into three components, namely, a gap due to the difference in the composition of characteristics (i.e., explained/endowment), a gap due to the difference in the effect of the characteristics (i.e., unexplained/coefficient), and a gap due to the interaction between the two components, endowment (i.e., explained part and coefficients, unexplained part).

**Table 3:** Detailed decomposition of CBHI enrollment by place of residence among households in Ethiopia using EMDHS 2019.

Decomposition	Coefficient with 95% CI	Percent explained	*p*-value
Row difference (R)	0.1257 (0.0928, 0.1586)	100	0.000
Explained (E)	0 0.0465 (0.0266, 0.0663)	36.98	0.000
Unexplained (C)	0.0792 (0.0420, 0.1164)	63.02	0.000

Covariates	Category	Coefficient with 95% CI	Percent	*p*-value
Endowment (Explained component) = Difference in characteristics (E)				
Sex of household head	Male	0.0020(−0.0011, 0.0050)	1.55	0.217
	Female	1	1	
Household head education	No education	1	1	
	Primary	0.0006(0.0002, 0.0009)	0.448	**0.002**
	Secondary	0.0071(0.0011, 0.0130)	5.612	**0.020**
	Higher	0.0185(0.0120, 0.0250)	14.718	**0.000**
Age of household head	≤24	1	1	
	25–64	0.0011(0.0002, 0.0019)	0.836	**0.012**
	≥65	0.0033(0.0005, 0.0062)	2.649	**0.023**
Family size	≤4	−0.0030(−0.0068, 0.0008)	−2.364	0.127
	5–6	0.0015(0.0002, 0.0028)	1.194	**0.023**
	≥7	1	1	
Number of under-five children	No child	1	1	
	1–2 children	0.0008(−0.0010, 0.0025)	0.614	0.388
	≥3 children	−0.0017(−0.0029, −0.0004)	−1.339	**0.008**
Wealth status	Poor	1	1	
	Middle	0.0123(0.0071, 0.0176)	9.812	**0.000**
	Rich	−0.0384(−0.0496, −0.0272)	−30.562	**0.000**
Region	Agricultural	1	1	
	Pastoralist	0.0024(0.0018, 0.0031)	1.942	**0.000**
	City	0.0389(0.0306, 0.0471)	30.941	**0.000**
Unexplained (due to difference in coefficients (C))				
Sex of household head	Male	0.0118(−0.1066, 0.1302)	9.371	0.845
	Female	1	1	
Household head education	No education	1	1	
	Primary	−0.0029(−0.0705, 0.0648)*	−2.281	0.934
	Secondary	0.0112(−0.0362, 0.0586)	8.927	0.643
	Higher	−0.0434(−0.1005, 0.0136)	−34.567	0.135
Age of household head	≤24	1	1	
	25–64	0.1150(−0.1353, 0.3654)	91.541	0.368
	≥65	0.0030(−0.0345, 0.0404)	2.378	0.876
Family size	≤4	0.0241(−0.1229, 0.1711)	19.172	0.748
	5–6	−0.0156(−0.0760, 0.0447)	−12.427	0.612
	≥7	1	1	
Number of under-five children	No child	1	1	
	1–2 children	0.0136(−0.0485, 0.0757)	10.793	0.669
	≥3 children	−0.0001(−0.0141, 0.0139)	0.0987	0.986
Wealth status	Poor	1	1	
	Middle	0.0102(−0.0105, 0.0310)	8.155	0.334
	Rich	0.1897(−0.0712, 0.4506)	150.92	0.154
Region	Agricultural	1	1	
	Pastoralist	−0.0266(−0.0529, −0.0003)	−21.179	**0.047**
	City	−0.0866(−0.16455, −0.0086)	−68.869	**0.030**

*The positive coefficient suggests that if rural households were brought to the same level of characteristics distribution as urban households, there would be a reduction in the disparity of CBHI enrollment rates between rural and urban areas. The bold value indicated that there is a significance at *p*-value <0.05.

The overall decomposition result showed that there is a significant disparity in the CBHI enrollment rate between urban and rural households (0.126, *p* < 0.001). The study found that 36.98% (*p* < 0.001) of the CBHI enrollment rate disparities were explained by the differences in the distribution of characteristics (endowments (E)) of households between urban and rural households, whereas we found that the differences in the effects (coefficients) of characteristics account for 63.02% (*p* < 0.001) of the observed rural–urban differences in the CBHI enrollment rate.

Table 3 also presents the detailed decomposition of the contribution of individual characteristics to the CBHI enrollment rate disparity between urban and rural households due to endowment and coefficients. The majority of the gap in CBHI enrollment was explained by the difference in wealth status between urban and rural households. The middle wealth status (9.81%) contributed to the narrowing of the gap, meaning if the composition of middle wealth status of the households was equalized between urban and rural households, the gap in CBHI enrollment rate would be narrowed by 9.81%, and rich wealth status (−30.56%) contributed to the widening of this gap.

Similarly, factors such as the distribution of age of household heads of 25–64 years (0.84%) and ≥ 65 years (2.65%), educational status of household heads with primary education (0.45%) and higher education (14.72%), family size of household 5–6 members (1.20%), and the household region category being city administration (30.94%) and pastoralist (1.94%) contributed to the narrowing of the gap in CBHI enrollment across rural and urban areas. This indicates that equalizing the composition of these characteristics between urban and rural households would be expected to reduce the gap in CBHI enrollment rates by 0.84% for household heads aged 25–64 years, 2.65% for household heads aged ≥65 years, 14.72% for household heads with higher education, and 31% for households in different regions (Table 3). Conversely, households with heads having secondary education (−5.62%) and households with 3 or more under-five children (−1.34%) contribute to widening the gap in CBHI enrollment rates across rural and urban households in Ethiopia (Table 3).

## Discussion

This study aimed to assess the rural–urban disparity of CBHI enrollment rate in Ethiopia using data from the EMDHS 2019 and the logit-based decomposition analysis. The finding of this study revealed that there was a significant disparity in CBHI enrollment between urban and rural households. We found that 36.98% of the observed rural–urban CBHI enrollment differentials were attributed to differences in household characteristics, and 63.02% of the observed differences were attributed to differences in the effect of household characteristics between rural and urban households. The previous study conducted on CBHI enrollment using spatial analysis supported this finding, revealing that there were geographical variations, such as urban and rural variations, in CBHI enrollment in Ethiopia (7). This implied that there is a need for targeted interventions to address the significant disparity in CBHI enrollment between urban and rural households. Policymakers, health planners, and healthcare managers should focus on equalizing CBHI enrollment among rural and urban households by ensuring the accessibility of the CBHI scheme in both urban and rural areas (4, 24, 30).

This study also found that the secondary and higher education levels of the household heads were the significant factors contributing to the reduction in urban–rural disparity of CBHI enrollment approximately by 6 and 15%, respectively. This finding implies that higher education among household heads plays a crucial role in narrowing the gap between urban and rural areas in terms of CBHI enrollment. When household heads have higher education levels, it positively impacts CBHI enrollment rates, potentially leading to more equitable access to healthcare services. Thus, policymakers and health planners should prioritize efforts to improve education levels, particularly among household heads in rural areas. Investing in education and expanding educational opportunities can help empower individuals to make informed decisions about healthcare, including enrolling in the CBHI scheme (7, 31, 32).

The number of under-five children was also found to be a significant contributing factor in the widening of the urban–rural disparity of CBHI enrollment rate by 1.34%. The finding implies that there is a need to have focused policies considering the number of under-five children in CBHI enrollment-related strategies and activities. Policymakers should prioritize family planning and reproductive health programs to empower individuals and families to make informed decisions. Additionally, strengthening maternal and child healthcare services in rural areas can help alleviate the burden on families and increase CBHI enrollment rates. Targeted interventions and support for families with multiple children, alongside addressing broader socioeconomic challenges, are essential to narrowing the gap and ensuring equitable access to healthcare services for all (31, 32).

Similarly, the study stated that the rich wealth status of the households significantly contributed to the widening of the disparity in CBHI enrollment by 30.56% between urban and rural households. This finding is supported by the study conducted on multidimensional determinants of CBHI in Ethiopia, which revealed that socioeconomic status was the determinant of CBHI enrollment (33). The finding implies the need for targeted interventions, improved financial accessibility, efforts to reduce income inequality, and strengthened social protection programs. Health decision-makers should prioritize strategies that address the specific needs of disadvantaged households, such as implementing subsidy programs and premium waivers, promoting inclusive economic growth, and providing targeted support to vulnerable populations. By addressing wealth disparities and enhancing access to healthcare services, policymakers can work toward narrowing the wealth-related disparity and ensuring equitable CBHI enrollment for households across both urban and rural areas (34–36).

### Policy and practical implications

The results of the study on the rural–urban disparity in CBHI enrollment in Ethiopia have important practical and policy implications. Policymakers and health managers should focus on addressing the disparities in enrolling for CBHI programs and strive to achieve equal enrollment rates across urban and rural households. Moreover, the study highlights the role of administrative regions in contributing to the rural–urban disparity in CBHI enrollment. This can help policymakers identify regions with lower enrollment rates and allocate resources equitably. It is important to address regional disparities and ensure that CBHI programs are accessible and effective across all regions of the country.

### Limitations of the study

The limitation of this study is its reliance on data from the EMDHS 2019, which may not reflect the current status of the urban–rural disparity in CBHI enrollment in Ethiopia as of 2024.

## Conclusion

The study found that there was a significant disparity in CBHI enrollment rate between urban and rural households in Ethiopia in 2019. Factors such as the age of household heads, educational level of household heads, family size, number of under-five children, region of the household, and wealth status of the household contributed significantly to the observed disparities due to the differentials in endowments of the households, and region of the households was the significant contributing factor for the disparities attributed to the effect of characteristics (coefficients).

## Data availability statement

The original contributions presented in the study are included in the article/supplementary material, and further inquiries can be directed to the corresponding author.

## Ethics statement

Ethical approval was not required for our study as we used the demographic and health survey, which identifies all data before making it public, and the DHS datasets we used are openly accessible. We obtained an authorization letter from CSA to download the DHS dataset at https://dhsprogram.com/. The dataset and all methods and data of demographic and health survey were performed based on DHS research guidelines.

## Author contributions

YT: Writing – review & editing, Writing – original draft, Visualization, Supervision, Software, Methodology, Investigation, Formal analysis, Data curation, Conceptualization. HA: Writing – review & editing, Methodology, Conceptualization. DG: Writing – review & editing, Methodology. AH: Writing – review & editing, Methodology. MJ: Writing – review & editing, Supervision, Methodology. KA: Writing – review & editing, Writing – original draft, Supervision. MT: Writing – review & editing, Supervision. KD: Writing – review & editing, Methodology. LA: Writing – review & editing, Methodology. AE: Data curation, Software, Writing – original draft, Writing – review & editing. WN: Writing – review & editing, Data curation. AW: Writing – review & editing, Data curation. LY: Writing – review & editing, Data curation. MG: Writing – review & editing, Data curation. NW: Writing – review & editing, Writing – original draft, Conceptualization. AB: Conceptualization, Writing – review & editing, Writing – original draft.
